# A framework to integrate artificial intelligence training into radiology residency programs: preparing the future radiologist

**DOI:** 10.1186/s13244-023-01595-3

**Published:** 2024-01-17

**Authors:** Maria Jorina van Kooten, Can Ozan Tan, Elfi Inez Saïda Hofmeijer, Peter Martinus Adrianus van Ooijen, Walter Noordzij, Maria Jolanda Lamers, Thomas Christian Kwee, Rozemarijn Vliegenthart, Derya Yakar

**Affiliations:** 1 Department of Radiology, Medical Imaging Center, University Medical Center Groningen, University of Groningen, PO Box 30.001, 9700 RB Groningen, The Netherlands; 2 Robotics and Mechatronics Group, Faculty of Electrical Engineering, Mathematics, and Computer Science, University of Twente, PO Box 217, 7500 AE Enschede, The Netherlands; 3 Department of Radiation Oncology, University Medical Center Groningen, University of Groningen, PO Box 30.001, 9700 RB Groningen, The Netherlands; 4 Machine Learning Lab, Data Science Center in Health (DASH), University Medical Center Groningen, University of Groningen, PO Box 30.001, 9700 RB Groningen, The Netherlands; 5 Department of Nuclear Medicine and Molecular Imaging, Medical Imaging Center, University Medical Center Groningen, University of Groningen, PO Box 30.001, 9700 RB Groningen, The Netherlands

**Keywords:** Artificial intelligence, Curriculum, Medical informatics, Training, Residency

## Abstract

**Objectives:**

To present a framework to develop and implement a fast-track artificial intelligence (AI) curriculum into an existing radiology residency program, with the potential to prepare a new generation of AI conscious radiologists.

**Methods:**

The AI-curriculum framework comprises five sequential steps: (1) forming a team of AI experts, (2) assessing the residents’ knowledge level and needs, (3) defining learning objectives, (4) matching these objectives with effective teaching strategies, and finally (5) implementing and evaluating the pilot. Following these steps, a multidisciplinary team of AI engineers, radiologists, and radiology residents designed a 3-day program, including didactic lectures, hands-on laboratory sessions, and group discussions with experts to enhance AI understanding. Pre- and post-curriculum surveys were conducted to assess participants’ expectations and progress and were analyzed using a Wilcoxon rank-sum test.

**Results:**

There was 100% response rate to the pre- and post-curriculum survey (17 and 12 respondents, respectively). Participants’ confidence in their knowledge and understanding of AI in radiology significantly increased after completing the program (pre-curriculum means 3.25 ± 1.48 (SD), post-curriculum means 6.5 ± 0.90 (SD), *p*-value = 0.002). A total of 75% confirmed that the course addressed topics that were applicable to their work in radiology. Lectures on the fundamentals of AI and group discussions with experts were deemed most useful.

**Conclusion:**

Designing an AI curriculum for radiology residents and implementing it into a radiology residency program is feasible using the framework presented. The 3-day AI curriculum effectively increased participants’ perception of knowledge and skills about AI in radiology and can serve as a starting point for further customization.

**Critical relevance statement:**

The framework provides guidance for developing and implementing an AI curriculum in radiology residency programs, educating residents on the application of AI in radiology and ultimately contributing to future high-quality, safe, and effective patient care.

**Key points:**

• AI education is necessary to prepare a new generation of AI-conscious radiologists.

• The AI curriculum increased participants’ perception of AI knowledge and skills in radiology.

• This five-step framework can assist integrating AI education into radiology residency programs.

**Graphical Abstract:**

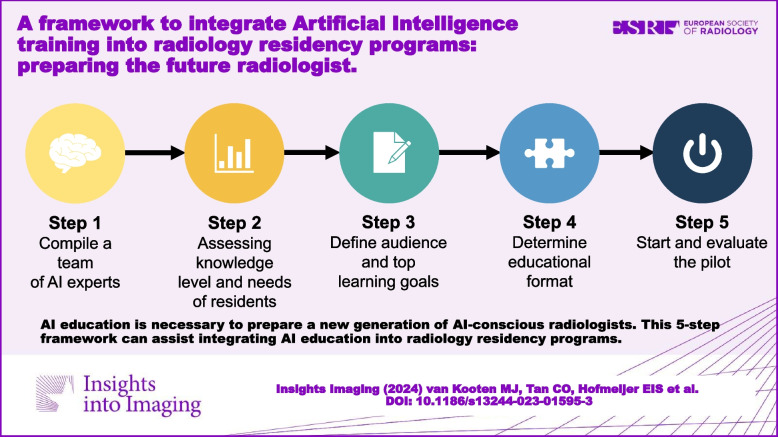

**Supplementary Information:**

The online version contains supplementary material available at 10.1186/s13244-023-01595-3.

## Introduction

The impact of artificial intelligence (AI) on healthcare is immense, with numerous applications already transforming clinical practice [1–3]. In radiology departments, the use of AI can improve administrative workflow, image acquisition, interpretation, and disease detection, transforming the role of radiologists in the process [4]. For example, AI-based algorithms can optimize radiology department workflows by prioritizing chest x-rays, thereby reducing report turnaround times for critical findings [5]. They also maximize image acquisition, such as by reducing noise and artifacts in MRI scans [6], and improve early detection of breast cancer in digital mammography [7]. A demonstration of a future workflow, illustrating how AI can impact multiple steps along the imaging life cycle, integrates various examples of how AI may assist [8]. Traditionally, radiologists are expected to make management decisions and investments in medical imaging equipment, picture archiving and communication systems, and other radiology information systems. However, the emergence of AI-powered diagnostic decision-making and workflow efficiency tools presents a new challenge that requires radiologists to have a basic understanding of AI systems [9]. Educating radiologists on the capabilities and limitations of AI, empowering them to assess AI systems, is crucial to ensure they can make optimal use of AI solutions to improve and sustain high-quality, safe, and effective patient care [10, 11].

While the importance of AI is inevitable, previous studies on developing an AI curriculum for existing radiology residency programs are scarce [12, 13]. Moreover, there is a general lack of formal AI training in radiology residency programs, and most academic institutions do not yet offer such training [14]. Therefore, we aim to present a framework to develop and implement a fast-track AI curriculum into an existing radiology residency program, with the goal of preparing a new generation of AI conscious radiologists.

## Methods

The Institutional Review Board of the University Medical Center Groningen (UMCG) approved the study, and informed consent was provided by the participants. Procedures followed were in accordance with the ethical standards and human regulations. This study is part of a subsidized project B3CARE (B3CARE; www.b3care.nl).

**Fig. 1:**
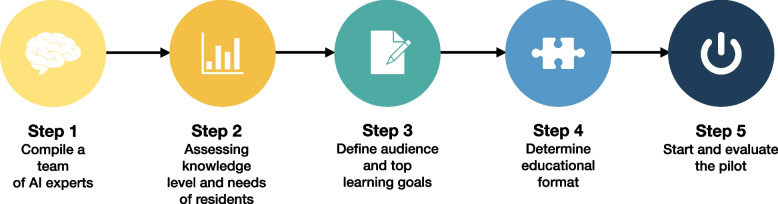
A five-step framework to develop and implement an AI curriculum into an existing radiology residency program. AI, artificial intelligence

We conducted a study on how to design, implement, and evaluate an AI curriculum, specifically tailored for educating radiology residents and emphasizing the assessment of its feasibility and endorsement of the radiology residents. In accordance, we developed a framework consisting of five sequential steps (Fig. 1).

1. Compilation of a multidisciplinary team of AI experts
2. Assessment of the knowledge levels and needs of the residents
3. Definition of learning goals
4. Matching these learning goals with effective methods
5. Execute and evaluate the pilot

Each step is described in more detail below. Following this five-step framework, a 3-day AI curriculum was designed by a multidisciplinary team of AI engineers, radiologists, and a radiology resident.

### Step 1: Compile a team of AI experts

A multidisciplinary team was formed to design and implement an AI curriculum in the radiology residency program. The team was composed of AI engineers with educational as well as scientific expertise from the Robotics and Mechatronics Group at the University of Twente (C.O.T., E.I.S.H.), data science experts as part of the Data Science Center in Health (DASH, UMCG, P.M.A.v.O., R.V.), radiologists with ample experience in the field of AI and current experience with the latest scientific advancements and practical applications from the Department of Radiology of the UMCG (D.Y., R.V., T.C.K.), radiology residency program directors (W.N., M.J.L.), and a radiology resident with special interest in AI in radiology (M.J.v.K.).

### Step 2: Ask your residents

A pre-curriculum survey (Supplementary material 1) was completed anonymously by all 17 residents including junior and senior residents (experience 1–5 years) working at the Radiology Department of the University Medical Center Groningen at April 2021. The pre-curriculum survey comprised a set of eight questions to inquire about residents’ current knowledge of AI, what specific topics they would like to learn about, and which learning methods they preferred. This information was then used in the designing and evaluation process of the AI curriculum.

### Step 3: Define audience and top learning goals

The optimal group size for the course was determined to be 12 participants based on various factors including residents’ preferences, practical considerations, and in order to facilitate effective interaction and group discussions. However, due to limited availability and a total of 17 radiology residents, priority was given to senior residents (3–5 years’ experience) over junior residents (1–2 years’ experience) by the radiology residency program directors and the AI expert team. Consequently, 11 senior residents with 3–5 years of experience (all senior residents working at the radiology department at October 2022) were included for participation in the course. Participation for the course was mandatory for the senior residents, and no prior skills or experience in computer science or programming were required. With one remaining vacancy, an attending radiologist with 10 years of working experience expressed interest in AI education and occupied the final seat. All participants were expected to have basic knowledge, understanding, and experience in medical imaging techniques and their application in clinical practice.

Learning goals were distilled by the AI expert team based on institutional goals and the results of the pre-curriculum survey among all residents. The top 3 learning goals were to understand the following: fundamental architecture structure of AI systems, how to exploit AI for clinical research, and how to use AI in clinical practice. These learning goals were defined in short contents: learning the fundamentals of AI, getting experience with building an algorithm, and understanding implication of AI in clinical research and clinical practice. An additional learning goal was to get familiar with billing, legal, and ethical aspects of AI use. See Table 1 for a more detailed description of the learning goals and Table 2 for a short description of the content. The learning goals of the present course were developed based on an established long-term curriculum designed for technical medicine students, which was a large-scale project supported by B3CARE. Our team of AI experts previously developed a relevant full-term course for technical medical students, providing a foundational structure that was condensed and modified to serve radiology residents in training.

**Table 1:** Learning goals distilled based on the institutional goals and the results of the pre-curriculum survey

Learning goals		
1	Follow fundamental architecture, structure, and implementation of AI systems to gain hands-on experience	This includes gaining experience on the following:
		a) The utility of AI in screening and triage
		b) The role of AI in precision medicine
		c) The limitations of the use AI in clinical practice
2	Understand how AI and machine learning approaches can be exploited for clinical research and associated shortcomings	These include recognition of the following:
		a) The significance of image quality and number of observations for effective AI application
		b) Consequences of using multi-institutional/multi-scanner data for research
		c) Overfitting, superfluous results, and their consequences
3	Broadly understand types of existing major open-source and commercial platforms that are already in use in clinical practice	This includes understanding and explain the following:
		a) The types and capabilities of commercially available AI systems and systems based on machine learning approaches
		b) How these can offer diagnostic support, such as image classification/detection, image segmentation, image registration, anomaly detection, and cross modality synthesis
		c) How they can integrate these systems into their clinical workflow to reduce burden on radiologists

*AI* artificial intelligence

**Table 2:** Short description of the contents of the AI curriculum divided over 3 days

Short contents			
1	Basics underlying AI algorithms	Including training procedures and assessment of the quality of fit (e.g., hyperparameters, overfitting)	Day 1
2	Fundamentals underlying AI algorithms	Assessment of accuracy, sensitivity, specificity, and other metrics of performance evaluation	Day 1
3	Quantitative approaches	To understand image quality and its impact	Day 1
4	Case presentations of use of AI in clinical research	Hypothesis versus data-driven approach, associations, and statistics	Day 2
5	Case presentations of use of AI in clinical care	Screening, triage, personalized medicine, and limitations of AI	Day 2
6	Overview of available commercial software	And their use cases	Day 3
7	Demonstration of basic integration of AI	Commercially available software to daily clinical workflow	Day 3
8	Discussions about billing, legal, and ethical issues	Group discussions about business models, insurance, law, and ethics	Day 3

*AI* artificial intelligence

### Step 4: Educational format

The learning goals of the curriculum were matched with different learning methods based on the pre-curriculum survey and the input of the AI expert team. The course format included didactic lectures about the basics of the underlying AI algorithms, hands-on laboratory sessions with building an algorithm on a clinical research data adopted from Orange (https://orangedatamining.com) (e.g., defining training and test sets, adjusting hyperparameters, consequences of overfitting, interpreting results), group discussions (billing, legal and ethical issues), Q/A sessions with an AI expert, and a presentation from a representative of a commercially available vendor. We asked the vendor to focus on providing insights on the integration of commercially available AI systems in institutional workflows and the associated implementation challenges, rather than marketing their software. Vendor’s presentation tools were reviewed by the instructor (C. O. T.) prior to the presentation, and the vendor clearly disclosed his potential conflict of interest.

The length of the curriculum was determined based on residents’ preferences (pre-curriculum survey results), feasibility of achieving learning goals, and practical feasibility of incorporation into the radiology residency program: the total length of the course was 3 days (8 h a day during regular working hours). Of these 24 h, roughly 9 h were dedicated towards the first learning goal, 9 to the second, and 6 h to the third.

### Step 5: Start and evaluate the pilot

A post-curriculum survey (Supplementary material 2) was completed by all 12 participants of the course (11 senior radiology residents and 1 experienced radiologist). The post-curriculum survey comprised a set of 22 questions aimed at evaluating the extent to which the participants’ expectations and learning goals were achieved, the influence of the course on their knowledge and skills pertaining to AI, and the potential impact of the course on their future career and professional work. Change in perceived confidence levels of participants before and after the course was rated retrospectively during the post-curriculum survey. Suggestions resulting from the post-curriculum survey and the insights of the AI expert team were collected for future revision of the curriculum. The development, running and evaluation of the AI curriculum utilizing the 5-step framework required a duration of approximately 20 months, with an estimated time commitment of 2 h per week in total.

### Statistical analysis

All results were analyzed descriptively. A Wilcoxon rank-sum test was conducted to evaluate the self-reported levels of confidence of the participants on knowledge and understanding of AI-based approaches in radiology before and after the curriculum, using R language for statistical computing.

## Results

**Fig. 2:**
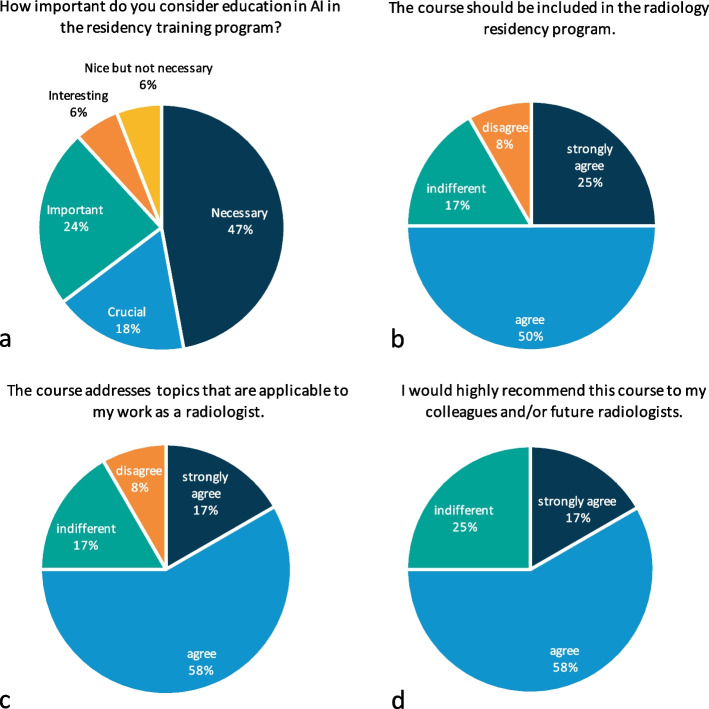
Highlighted results of the pre-curriculum and post-curriculum survey. *0 values are not shown on the pie charts. **a** Question from the pre-curriculum survey including 17 responses. **b**–**d** Questions from the post-curriculum survey including 12 responses per question

A total of 17/17 residents completed the pre-curriculum survey (100% response rate). The survey indicated that most residents had little to no knowledge or experience with AI (Table 3). Most residents believed it was necessary to implement AI education in the radiology residency program (Fig. 2). Topics that residents wanted to be included in the curriculum were as follows: how to implement AI in the radiologist’s workflow (15 residents/88.2%), understanding machine learning and deep learning (7 residents/41.2%), and how AI can be used in clinical practice (12 residents/70.6%) and for research purposes (10 residents/58.8%) (Table 3). Residents suggested various learning methods: integrating education in AI into the existing clinical rotations (10 residents/58.8%), a separate learning course about AI (9 residents/52.9%), or an online learning module about AI (8 residents/47.1%) (Table 3).

**Table 3:** Results from the pre-curriculum survey consisting of eight questions

Pre-curriculum survey (*n* = 17 residents)		
**In which hospital did you originally enroll for the radiology residency program?**	Academic hospital	12 (70.6%)
	Nonacademic hospital	5 (29.4%)
**What is the extent of your knowledge and experience in the field of AI?**	No experience with AI	1 (5.9%)
	Heard about AI	11 (64.7%)
	Had some lectures about AI	8 (47.1%)
	Engaged with AI	6 (35.3%)
**How important do you consider education in AI in the radiology residency program?**	Crucial	2 (17.6%)
	Necessary	8 (47.1%)
	Important	4 (23.5%)
	Interesting	1 (5.9%)
	Fine	1 (5.9%)
	Not necessary	0 (0%)
**Which specific topics would you prioritize for inclusion in the course (multiple options possible)?**	How to implement AI in the workflow of the radiologist	15 (88.2%)
	Understanding about machine learning and deep learning	7 (41.2%)
	How can AI be used in clinical practice	12 (70.6%)
	How can AI be used for research purposes	10 (58.8%)
**Which learning method would you suggest for the course (multiple options possible)?**	Integration education in AI into the clinical rotations	10 (58.8%)
	Separate learning course	9 (52.9%)
	Online learning module	8 (47.1%)
**What duration do you recommend for the course?**	Continuous time to the radiology residency program	1 (5.9%)
	longer than 1 month	4 (23.5%)
	1 month	4 (23.5%)
	3 weeks	1 (5.9%)
	1 week	6 (35.3%)
**How much time are you willing to devote to self-study and coursework outside of regular working hours?**	Only during regular working hours	3 (17.6%)
	1 day	3 (17.6%)
	1 week	6 (35.3%)
	1 month	4 (23.5%)
	As long as needed	1 (5.9%)
**Do you have any suggestions or recommendations for the course?**	Comment: I feel that there is too much repetition in discussions about neural networks and how they work. I am particularly interested in information regarding the clinical and research applications of AI. Experts in this field can provide guidance and fully comprehend the details of neural networks. I think it would be a stretch for all radiologists/residents to become experts in this area, but basic knowledge seems appropriate Do’s: Teach specific terminology so that residents can independently read and critique AI-related articles. Provide an overview of data augmentation techniques to enhance the robustness of the algorithm, such as duplicating, rotating, and flipping the training set Don’ts: Analyze the CNN architecture in detail. In my opinion, this adds little value to the concept, and most physicians will not absorb enough information to retain it Comment: It is especially important that we understand these concepts for the future. As AI becomes more prevalent, we will need to know more about it to assume a more supervisory role. Therefore, what will our role be in this?	

*AI* artificial intelligence, *CNN* convolutional neural network. Seventeen residents working at the radiology department with 1–5 years of experience responded to the survey

**Fig. 3:**
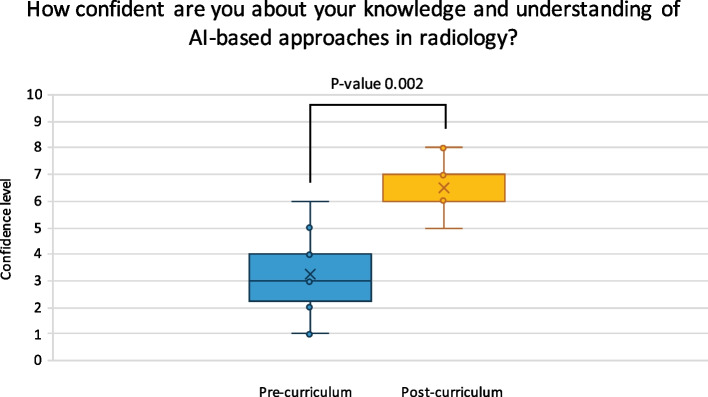
Level of confidence of the participants about their knowledge and understanding of AI-based approaches in radiology. AI, artificial intelligence. A total of 12 responses on confidence level of participants on a scale from 0 to 10 (0 = not confident at all, 10 = very confident) that they rated during the post-curriculum survey. A Wilcoxon rank-sum test resulted in a pre-curriculum mean of 3.25 (*SD* = 1.48) and post-curriculum mean of 6.5 (*SD* = 0.90), *Z*-value =  − 3.06 and *p*-value = 0.002

**Fig. 4:**
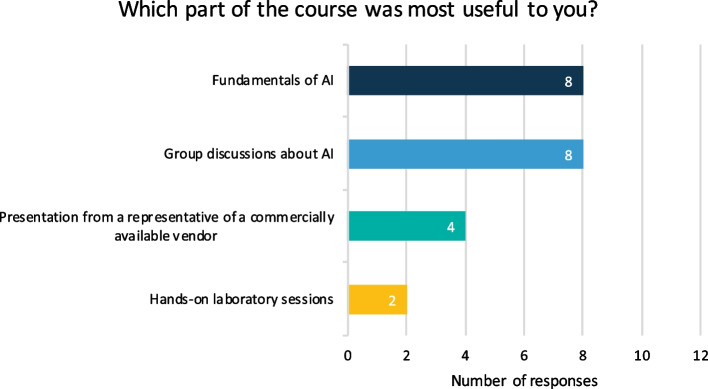
Results on which part of the AI curriculum was evaluated most useful. AI, artificial intelligence. Question from the post-curriculum survey including 12 responses; multiple options were possible

A total of 12/12 participants completed the post-curriculum survey (100% response rate). After completion, participants’ rated confidence levels on knowledge and understanding of AI-based approaches in radiology were significantly increased (retrospectively rated pre-curriculum mean of confidence level = 3.25 (*SD* = 1.48) and post-curriculum mean of confidence level = 6.5 (*SD* = 0.90), *Z*-value =  − 3.06, *p*-value = 0.002) (Fig. 3). The group size (12 participants) and time investment (3 days) for the course were deemed efficient (Table 4). Moreover, 9 participants (75%) confirmed that the course covered topics that were applicable to their work as radiologists (Fig. 4). Lectures on the fundamentals of AI and group discussions on AI were rated most useful (Fig. 4). Only 2 participants (16.7%) found the hands-on laboratory sessions (building an algorithm on pre-defined clinical research data) useful (Table 4). Nine participants (75%) noted that they would highly recommend the course to their colleagues and future radiologists, and 9 participants (75%) were positive about including the course in the regular radiology residency program (Fig. 4). However, the course was found to cover certain areas insufficiently, including hospital management’ views on legal and insurance issues related to AI, input from radiologists that integrated AI in their workflow (for example: how they select an appropriate AI software), and cost-effectiveness strategies of AI-powered tools in healthcare in Europe (Table 4).

**Table 4:** Results from the post-curriculum survey consisting of 22 questions

Post-curriculum survey (*n* = 12 participants)		
What is your function at work?	Radiology resident	11 (91.7%)
	Radiologist	1 (8.3%)
What year of residency or experience are you at?	3	5 (41.7%)
	4	3 (25%)
	5	3 (25%)
	10	1 (8.3%)
How confident were you about your knowledge and understanding of AI-based approaches in radiology before the AI course? (Scales 1–10 with 1 = not confident at all and 10 = very confident)	1	2 (16.7%)
	2	1 (8.3%)
	3	4 (33.3%)
	4	3 (25%)
	5	1 (8.3%)
	6	1 (8.3%)
	7	0 (0%)
	8	0 (0%)
	9	0 (0%)
	10	0 (0%)
The course information increased my knowledge and skills about AI	Strongly disagree	0 (0%)
	Disagree	1 (8.3%)
	Indifferent	1 (8.3%)
	Agree	9 (75%)
	Strongly agree	1 (8.3%)
The course gave me more confidence on how to evaluate new AI projects	Strongly disagree	0 (0%)
	Disagree	0 (0%)
	Indifferent	7 (58.3%)
	Agree	4 (33.3%)
	Strongly agree	1 (8.3%)
After the course, I understand more about the shortcomings and strengths of AI	Strongly disagree	0 (0%)
	Disagree	0 (0%)
	Indifferent	2 (16.7%)
	Agree	9 (75%)
	Strongly agree	1 (8.3%)
The course addresses topics that are applicable to my work as a radiologist	Strongly disagree	0 (0%)
	Disagree	1 (8.3%)
	Indifferent	2 (16.7%)
	Agree	7 (58.3%)
	Strongly agree	2 (16.7%)
Which part of the course was most useful to you (multiple options are possible)?	Fundamentals of AI	8 (66.7%)
	Hands-on laboratory sessions	2 (16.7%)
	Group discussions about AI	8 (66.7%)
	Presentation from a representative of a commercially available vendor	4 (33.3%)
Which topics did you find most interesting and/or useful?	Which topics did you find most interesting and/or useful? I thought the talks and discussions about how these things come to life were very insightful. Also, our speaker was great! The fundamentals of course were important to get a grasp of the general concepts. The hands-on laboratory sessions were not something I could now implement in daily research practice, but I suppose that was not necessarily the goal. The commercial vendor was a great addition because it shows real clinical application of AI and its potential, as well as its downsides/pitfalls. It did not feel like a sales pitch The fundamentals are not the most interesting but definitely extremely important. Self-practicing with a model is very insightful and would be great to do that more independent and compare outcomes of individuals afterwards. Practical application by vendor was great, since it was not about the product but how work will change through new techniques Fundamentals of AI	
The combination of theory and hands-on was useful	Strongly disagree	2 (16.7%)
	Disagree	1 (8.3%)
	Indifferent	4 (33.3%)
	Agree	3 (25%)
	Strongly agree	2 (16.7%)
The balance between theory, clinical application, and hands-on was well balanced	Strongly disagree	2 (16.7%)
	Disagree	1 (8.3%)
	Indifferent	2 (16.7%)
	Agree	7 (58.3%)
	Strongly agree	0 (0%)
What did you think about the group size (12 people)?	Good	10 (83.3%)
	Larger groups are more effective	1 (8.3%)
	12–15 people would be ideal	1 (8.3%)
What did you think about the length of the course (3 days)?	Good	7 (58.3%)
	Bit long	3 (25%)
	2 days would be more efficient and is possible	2 (16.7%)
The course was helpful in my progress towards my degree	Strongly disagree	0 (0%)
	Disagree	1 (8.3%)
	Indifferent	6 (50%)
	Agree	4 (33.3%)
	Strongly agree	1 (8.3%)
The course is likely to influence my radiology practice in the future	Strongly disagree	0 (0%)
	Disagree	1 (8.3%)
	Indifferent	5 (41.7%)
	Agree	5 (41.7%)
	Strongly agree	1 (8.3%)
After completing this course, how confident are you about your knowledge and understanding of AI-based approaches in radiology? (Scales 1–10 with 1 = not confident at all and 10 = very confident)	1	0 (0%)
	2	0 (0%)
	3	0 (0%)
	4	0 (0%)
	5	1 (8.3%)
	6	6 (50%)
	7	3 (25%)
	8	2 (16.7%)
	9	0 (0%)
	10	0 (0%)
I would highly recommend this course to my colleagues and/or future radiologists	Strongly disagree	0 (0%)
	Disagree	0 (0%)
	Indifferent	3 (25%)
	Agree	7 (58.3%)
	Strongly agree	2 (16.7%)
The course should be included in the radiology residency program	Strongly disagree	0 (0%)
	Disagree	1 (8.3%)
	Indifferent	2 (16.7%)
	Agree	6 (50%)
	Strongly agree	3 (25%)
All radiologists should follow this course to understand more about AI	Strongly disagree	0 (0%)
	Disagree	2 (16.7%)
	Indifferent	4 (33.3%)
	Agree	3 (25%)
	Strongly agree	3 (25%)
What would you recommend on how to improve AI knowledge in the radiology residency program (multiple options are possible)?	Lectures about AI from guest speakers	8 (66.7%)
	Journal clubs with the radiology department	1 (8.3%)
	Following online courses offered by radiology associations	4 (33.3%)
	Interdisciplinary conferences about implementing AI in clinical practice	5 (41.7%)
	Demos or simulations by AI companies	7 (58.3%)
	AI course with a small group	9 (75%)
	AI discussions with residents and/or radiologists	5 (41.7%)
	Discussions about ethics, financial, and insurance aspects of AI	4 (33.3%)
	Regional organized education	1 (8.3%)
Are there any topics of discussions you missed during the course and you would like to discuss?	Views from our hospital on the matter or the input of radiologists that work with AI: how did they select a program, what is their cost-effectiveness strategy, etc	
Do you have tips or ideas on how to improve this AI course?	Bit more hands-on. Maybe some easy examples with a little coding, do not know if that is feasible Communication beforehand should be much better Research part was not very useful to me During the UMCG resident day (for all residents, not just radiology residents) earlier this year, I attended a workshop offered by DASH in the UMCG during which we got to try out Google Teachable Machine (https://teachablemachine.withgoogle.com). With several small image datasets, we got to dabble with AI at a very basic level. Maybe this could also be implemented during the hands-on laboratory sessions of this course, as it is a very simple yet understandable way of showing how it works. Maybe afterwards, the Orange hands-on part is a bit easier to understand The hands-on laboratory sessions could be set up with more group participation; now it is as a loose part	

*AI* artificial intelligence, *DASH* Data Science Center in Health, *UMCG* University Medical Center Groningen. Eleven residents with 3–5 years of experience and 1 radiologist with 10 years of experience responded to the survey

## Discussion

With this study, we demonstrated the feasibility of implementing a framework for developing and delivering a 3-day AI curriculum in a radiology residency program. The framework consisted of five key steps (composing an AI expert team, assessing knowledge and needs of residents, defining audience and learning goals, determining educational format, and staring and evaluating a pilot) that other radiology departments may find useful to prepare a similar fast-track AI curriculum. The post-curriculum survey indicated that the curriculum improved participants’ self-reported confidence in how to handle AI-based approaches in radiology practice. Participants also recommended that the curriculum should be included as a standard component in the existing radiology residency program. Lectures on the fundamentals of AI and group discussions with experts were deemed most useful, while hands-on laboratory sessions with building an algorithm were rated as less useful. Furthermore, the participants perceived a course length of 3 days as sufficient. These results serve as a marker of the curriculum’s effectiveness, underlining its practical utility within radiology residency programs.

Two previous studies [12, 13] reported on the development and implementation of AI curriculum in radiology residency programs. Shiang et al. [12] evaluated residents’ real-time experience and perception of using AI-based decision support system applications. The residents found the approach desirable and reported positive experiences. However, a major limitation of their study was the lack of generalizability because they focused on the use of one commercially available platform rather than the concepts underlying AI-based systems and their practical application. Thus, this approach may not provide a thorough overview of the potential role of AI in radiology practice, and may not be applicable or appropriate for other institutions that use different platforms or vendors. Moreover, this study did not include a detailed description of learning goals on more foundational concepts of machine learning or deep learning. Nonetheless, we do agree that their design of training will prepare the participants for future AI advancements, and a real-time experience could be a part of a more comprehensive curriculum.

Hu et al. [13] developed a 3-week AI workshop. Their results on their post-workshop surveys showed increasing confidence in understanding AI concepts by the participating residents, similar to ours. They presented a comprehensive overview of methodology with learning objectives that make it more generalizable to other institutions. Nonetheless, a major limitation of this study is the length of the course (3 weeks), which limits the feasibility of embedding this course to radiology resident training programs. In our study, we designed a curriculum of 3 days, which the participants perceived as sufficient and the radiology residency program directors found feasible for easy incorporation into the existing radiology residency schedule. However, Hu et al. delved extensively into the technical aspects of AI, whereas we focused on broader aspects such as implementation, legislation, and ethics.

Recently, Salastekar et al. [15] highlighted the need for education in AI based on a survey among 759 residents in the USA. They found that a majority of radiology residents believed that education in AI should be included in the radiology training program. They found that hands-on laboratory sessions and didactic lectures were rated as the most effective learning methods but, in our study, especially the hands-on laboratory sessions, were not evaluated as most valuable. This may be because we used an open-source, research data set (Orange, https://orangedatamining.com). Some residents found this approach to be too technical (writing and adjusting algorithms), and preferred an easier and more visual method, which might be closer to the work of a radiologist. This is in line with the findings in other previous studies were residents felt AI to be important and worth learning, but most were not very interested in learning to program [16] or simplified, and self-contained coding environments could also serve as fertile opportunity for self-exploration [17]. However, preferences may not be the sole reasoning behind avoiding the technical intricacies. The team of AI experts still find the relative in-depth technical approach to be necessary. In future courses, balancing these perspectives, a less technical and a more visual approach, could be considered based on residents’ needs and expert recommendations.

Regarding the content, in our post-curriculum survey, participants felt that some topics were missing from the course. For example, input from radiologists currently working with AI, hospital management’ views on legal and insurance issues related to AI, and cost-effectiveness strategies of AI-powered tools in health care in Europe were not covered in our curriculum. Similar findings were reported by Huisman et al. [18] who surveyed 1041 radiologists on AI in radiology and concluded that AI education should include issues related to data management, ethics, and legislation. We addressed data management and implementation challenges by inviting a representative from a commercial vendor to provide insight during group discussions. This part of the curriculum can be further improved by including more (or specific) commercial AI software (Van Leeuwen et al. provided a full list of 100 commercially available AI software for radiology [19]), although this would add to the overhead by increasing the need for external resources and time commitment.

Ethical issues were also addressed during our sessions in the form of group discussions. However, we did not include an ethics expert with experience in clinical use of AI, and did not cover legislative aspects, hospital management views, or cost-effectiveness strategies in Europe. As these are emerging topics, it would be valuable to include group discussions with hospital management, policy makers, and insurance companies to discuss future challenges.

In our curriculum, we did not use any existing learning or vendor-based platforms. While some learning platforms are open source and can facilitate learning by providing access to the latest AI tools and resources, others come with a price tag. Depending on the budget and learning goals, the use of existing learning platforms could be useful while also allowing each institution to customize learning goals according to their own specific needs and practice.

Our study did not issue certificates upon course completion due to our non-accreditation status and the current lack of standardized certification for AI in healthcare. Recognizing the involvement of global entities in standardizing AI in healthcare, we suggest considering accreditation and skills evaluation as a next step for AI courses in the medical field. A recent study identified six competencies for physicians using AI tools in healthcare: foundational knowledge, critical appraisal, medical decision-making, technical use, patient communication, and awareness of unintended consequences [20]. A framework like this one could be used in shaping assessments for AI education of physicians.

This proposal presents some limitations that should be discussed. First, our findings are somewhat context dependent as different settings may have different needs and resources available. We compiled a team of AI experts, which may not be feasible in other institutions. Nonetheless, our institution does not have access to large platforms that are integrated into daily radiology practice yet, which may in fact be comparable to many institutions worldwide. Second, our self-reported pre- and post-assessment surveys may introduce bias by inflating participants’ confidence post-curriculum due to the positive experience of completing the curriculum. The absence of objective assessments to measure participants’ proficiency in understanding and evaluating AI software before and after the course is a limitation. To enhance future curricula, we recommend integrating more objective assessments pre- and post-curriculum, such as multiple-choice questions, case-based assessments, or practical exams, ensuring a more comprehensive evaluation of participants’ AI-related competence. Besides, in our surveys, we employed the Likert scaling method to evaluate various components of the curriculum. Additionally, integrating the net promoter score (NPS) may be beneficial, especially for queries related to recommending the curriculum. Third, our curriculum was developed and executed in a single academic hospital and with a small sample size (12 participants). Therefore, our results may not fully generalize to other radiology departments. However, its independence from a commercially available platform and its practical feasibility in terms of course duration would make it easier to implement in any residency program. Lastly, the implementation of the curriculum in a singular manner, combined with the reliance on a one-time post-curriculum survey, lacks a longitudinal assessment. Integrating an annually repeating curriculum accompanied by consistent survey results and experiential feedback would enhance its value. We plan to further personalize our AI curriculum to make it available annually to our radiology residents and to use annual survey’ results to further meet the needs of the participants continuously. Investigating these aspects further through a multicenter approach for future AI educational programs would be interesting. This could potentially offer a broader perspective and deeper insight into the perceptions and experiences of residents across different institutions.

In conclusion, designing an AI curriculum for radiology residents and implementing it into a radiology residency program is feasible using the framework presented. The 3-day AI curriculum effectively increased participants’ perception of knowledge and skills about AI in radiology and can serve as a starting point for further customization.

### Supplementary Information

**Additional file 1.** 

## Data Availability

All data generated or analyzed during this study are included in this published article.

## Abbreviations

AI: Artificial intelligence
CNN: Convolutional neural network
DASH: Data Science Center in Health
UMCG: University Medical Center Groningen
