# Supplementary material for: A framework to integrate artificial intelligence training into radiology residency programs: preparing the future radiologist
Source: Insights Imaging. 2024 Jan 17;15:15. doi: 10.1186/s13244-023-01595-3 (PMC10792147; doi:10.1186/s13244-023-01595-3)
Supplement: Supplementary file 1 — Additional file 1. [file 13244_2023_1595_MOESM1_ESM.docx]

**A framework to integrate Artificial Intelligence training into radiology residency programs: preparing the future radiologist.**

**ELECTRONIC SUPPLEMENTARY MATERIAL**

| Pre-curriculum survey consisting of 8 questions. | |
| --- | --- |
| 1. In which hospital did you originally enroll for the radiology residency program? | Academic hospital  Non-academic hospital |
| 1. What is the extent of your knowledge and experience in the field of AI? | No experience with AI  Heard about AI  Had some lectures about AI  Engaged with AI |
| 1. How important do you consider education in AI in the radiology residency program? | Crucial  Necessary  Important  Interesting  Fine  Not necessary |
| 1. Which specific topics would you prioritize for inclusion in the course (multiple options possible)? | How to implement AI in the workflow of the radiologist  Understanding about machine learning and deep learning  How can AI be used in clinical practice  How can AI be used for research purposes  Systems underlying AI  Machine learning and deep learning  Other |
| 1. Which learning method would you suggest for the course (multiple options possible)? | Integration education in AI into the clinical rotations  Separate learning course  Online learning module  Alone  With a group  Evening lectures  Workshop  Own project  Other |
| 1. What duration do you recommend for the course? | Continuous time to the radiology residency program  longer than 1 month  1 month  3 weeks  1 week  Other |
| 7. How much time are you willing to devote to self-study and coursework outside of regular working hours? | Only during regular working hours  1 day  1 week  1 month  As long as needed  Other |
| 8. Do you have any suggestions or recommendations for the course? | Open text |

AI; artificial intelligence.

| Post-curriculum survey consisting of 22 questions. | |
| --- | --- |
| 1. What is your function at work? | Radiology resident  Radiologist |
| 2. What year of residency or experience are you at? | Open text |
| 3. How confident were you about your knowledge and understanding of AI-based approaches in radiology before the AI course? (scale 1-10 with 1 = not confident at all and 10 = very confident) | Linear scale 1 to 10 |
| 4. The course information increased my knowledge and skills about AI. | Strongly disagree  Disagree  Indifferent  Agree  Strongly agree |
| 5. The course gave me more confidence on how to evaluate new AI projects. | Strongly disagree  Disagree  Indifferent  Agree  Strongly agree |
| 1. After the course I understand more about the shortcomings and strengths of AI. | Strongly disagree  Disagree  Indifferent  Agree  Strongly agree |
| 1. The course addresses topics that are applicable to my work as a radiologist. | Strongly disagree  Disagree  Indifferent  Agree  Strongly agree |
| 1. Which part of the course was most useful to you (multiple options are possible)? | Fundamentals of AI  Hands-on laboratory sessions  Group discussions about AI  Presentation from a representative of a commercially available vendor |
| 1. Which topics did you find most interesting and/or useful? | Open text |
| 1. The combination of theory and hands-on was useful. | Strongly disagree  Disagree  Indifferent  Agree  Strongly agree |
| 1. The balance between theory, clinical application, and hands-on was well balanced. | Strongly disagree  Disagree  Indifferent  Agree  Strongly agree |
| 1. What did you think about the group size (12 people)? | Open text |
| 1. What did you think about the length of the course (3 days)? | Open text |
| 1. The course was helpful in my progress towards my degree. | Strongly disagree  Disagree  Indifferent  Agree  Strongly agree |
| 1. The course is likely to influence my radiology practice in the future. | Strongly disagree  Disagree  Indifferent  Agree  Strongly agree |
| 1. After completing this course, how confident are you about your knowledge and understanding of AI-based approaches in radiology? (scale 1-10 with 1 = not confident at all and 10 = very confident) | Linear scale 1 to 10 |
| 1. I would highly recommend this course to my colleagues and/or future radiologists. | Strongly disagree  Disagree  Indifferent  Agree  Strongly agree |
| 1. The course should be included in the radiology residency program. | Strongly disagree  Disagree  Indifferent  Agree  Strongly agree |
| 1. All radiologists should follow this course to understand more about AI. | Strongly disagree  Disagree  Indifferent  Agree  Strongly agree |
| 1. What would you recommend on how to improve AI knowledge in the radiology residency program (multiple options are possible)? | Lectures about AI from guest speakers  Journal clubs with the radiology department  Following online courses offered by radiology associations  Interdisciplinary conferences about implementing AI in clinical practice  Demos or simulations by AI companies  AI course with a small group  AI discussions with residents and/or radiologists  Discussions about ethics, financial and insurance aspects of AI  Regional organized education  Other |
| 1. Are there any topics of discussions you missed during the course and you would like to discuss? | Open text |
| 1. Do you have tips or ideas on how to improve this AI course? | Open text |

AI; artificial intelligence.
